# Low-dose x-ray tomography through a deep convolutional neural network

**DOI:** 10.1038/s41598-018-19426-7

**Published:** 2018-02-07

**Authors:** Xiaogang Yang, Vincent De Andrade, William Scullin, Eva L. Dyer, Narayanan Kasthuri, Francesco De Carlo, Doğa Gürsoy

**Affiliations:** 1 X-ray Science Division, Argonne National Laboratory, 9700 South Cass Avenue, Lemont, IL 60439 USA; 2 Argonne Leadership Computing Facility (ALCF), Argonne National Laboratory, 9700 South Cass Avenue, Lemont, IL 60439 USA; 3 Department of Biomedical Engineering, Georgia Institute of Technology & Emory University, 313 Ferst Dr NW, Atlanta, GA 30332 USA; 4 Biology Division, Argonne National Laboratory, 9700 South Cass Avenue, Lemont, IL 60439 USA; 5 Department of Neurobiology, University of Chicago, 947 East 58th Street, Chicago, IL 60637 USA; 6 Department of Electrical Engineering and Computer Science, Northwestern University, 2145 Sheridan Road, Evanston, IL 60208 USA

## Abstract

Synchrotron-based X-ray tomography offers the potential for rapid large-scale reconstructions of the interiors of materials and biological tissue at fine resolution. However, for radiation sensitive samples, there remain fundamental trade-offs between damaging samples during longer acquisition times and reducing signals with shorter acquisition times. We present a deep convolutional neural network (CNN) method that increases the acquired X-ray tomographic signal by at least a factor of 10 during low-dose fast acquisition by improving the quality of recorded projections. Short-exposure-time projections enhanced with CNNs show signal-to-noise ratios similar to long-exposure-time projections. They also show lower noise and more structural information than low-dose short-exposure acquisitions post-processed by other techniques. We evaluated this approach using simulated samples and further validated it with experimental data from radiation sensitive mouse brains acquired in a tomographic setting with transmission X-ray microscopy. We demonstrate that automated algorithms can reliably trace brain structures in low-dose datasets enhanced with CNN. This method can be applied to other tomographic or scanning based X-ray imaging techniques and has great potential for studying faster dynamics in specimens

## Introduction

Beginning with the advent of X-ray computerized tomography (CT) for routine scanning back in the early 1970s^1^, X-ray CT has grown into a powerful imaging modality that can provide the internal three-dimensional (3D) morphology of representative volumes of biological tissues and material science specimens. With the use of appropriate X-ray optics for focusing the beam (e.g. zone-plates, capillaries, Kirkpatrick-Baez mirrors, etc.), reconstructing projections with tens of nanometers of spatial resolution is within reach. However, there is an inherent trade-off between signal-to-noise ratio (SNR) and beam damage when imaging at the nanoscale^2^ because the radiation dose absorbed by the sample scales inversely with the resolution^3^.

There are potential hardware and software solutions to compensate for beam damage induced by long-exposure acquisitions. Hardware solutions primarily include cryogenic cooling with integrated cryostages^4–8^. Inconveniently, such devices require operating under vacuum conditions and make it impossible to use high precision air-bearing rotary stages or to perform *operando* experiments using environmentally controlled cells to simulate real operational conditions (i.e., load, pressure, temperature, etc.). Moreover, even in cryogenic conditions, the amount of radiation dose deposited for resolving a 10 nm feature is estimated to be about 10^10^ Gray^9^, which is at the borderline of inducing irreversible damage for biological samples.

Computational solutions for improving low-dose CT data provide an alternative. Existing algorithmic approaches are essentially applied to the data after collection, either through denoising^10,11^, improving reconstruction algorithms^12–16^, or through other post-processing methods that are applied to the reconstructed images^17–19^. Because many of these approaches aim to denoise the data without knowledge of the structures of interest, they run the risk of either generating new artifacts in the data or losing structural information through post-processing. Thus, neither current hardware or software solutions provide a clear path to fast X-ray imaging of radiation sensitive samples at nanometer resolution.

A better approach is to enhance signals from low-dose projections during acquisition itself, avoiding the potential pitfalls described above. Deep learning methods, particularly convolutional deep neural networks (CNNs)^20^, are promising algorithmic approaches for addressing this issue. CNNs have been widely used for image denoising^21–23^, super-resolution microscopy^24–27^, and even *post-hoc* denoising of low-dose X-ray tomography reconstructions^18^. Despite their promise, CNNs have not yet been used to enhance acquisition data by learning corresponding ‘maps’ between features in low-dose and high-dose images of the same sample, and applying these learned maps to low-dose projections from the same sample. Since both the training and raw images are collected from the same dataset, it is unnecessary to estimate an additive noise model to correct the data.

In this article, we introduce a CNN-based approach for learning the mapping between a number of pairs of low-dose and high-dose projections. Using a limited number of high-dose training examples, we can then use the trained network to predict high-dose reconstructions from a full-rotation tomographic dataset with short-exposure times. The proposed approach can be applied to a range of low-dose tomography applications (e.g. lab-based CT systems); however, we test it using transmission x-ray microscopy (TXM)^28^. We applied the method to recover adult mouse brain structures (e.g. myelinated axon tracts) in 3D at nanometer length scales (∼50 nm) and successfully demonstrate that the CNN-based method can provide sufficient signal for automated tracing of individual axons. By combining computational imaging approaches with a multi-stage approach for tomography, we demonstrate that high-quality reconstructions can be obtained at a fraction of the radiation dose.

## Results

### Evaluation with synthetic data

**Figure 1:**
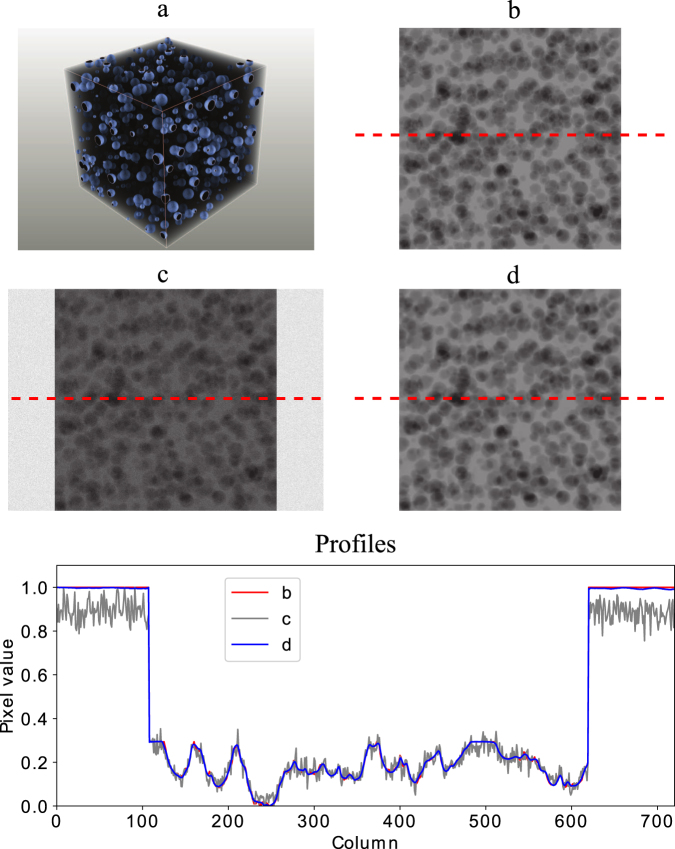
The simulation data to evaluate the CNN algorithm performance: (**a**) the synthetic phantom for the simulation, the empty space, the cubic and the particles have the gray values of 0, 0.3 and 1 respectively. The particle diameters are 4 to 20 voxels; (**b**) one of the original high-quality projections from the synthetic phantom, *P*(0); (**c**) the projection with 5% artificial noise, *P*_*n*05_. Result: (**d**) the enhanced projection by the CNN algorithm, *P*_*e*05_(0) showing the background noise perfectly removed and structural information in good agreement with (**b**).

To quantitatively evaluate improvements obtained using our CNN-based approach, we first created a synthetic dataset - a solid cube with 1000 sphere shaped particles randomly distributed throughout a 512 × 512 × 512 volume (see Fig. 1(a) for details). The synthetic dataset served as ground truth and allowed us to model different exposure conditions through adding noise to the data (e.g. shorter exposure times correspond to adding more noise). We generated 721 high-dose projections (*P*) from 0 to 180 degrees by applying a Radon transform to the object (Fig. 1(b)) and then added Gaussian noise (5% to 30%) to simulate low dose measurements at variable exposure time (Fig. 1(c)).

Using low- and high-dose pairs, we trained a CNN to estimate the high-resolution measurements (*P*_*o*_) when provided with low-dose projections (*P*_*n*_). Specifically we trained the CNN using the projection pairs at 0° (*P*_*o*_(0) and *P*_*n*_(0)) and 45° (*P*_*o*_(45) and *P*_*n*_(45)) for all noise levels: (*f*: (*P*_*o*_(0), *P*_*o*_(45)) → (*P*_*n*_(0), *P*_*n*_(45))). To obtain a mean square error between the predicted projections and ground truth dataset of less than 10^−4^ (40 epochs), CNN training took about 2 hours (213 *s* × 40 epochs) with a Nvidia Quadro M5000 GPU. The trained CNN is used to process the full angle *P*_*n*_ to obtain the enhanced projections *P*_*e*_. Our results on synthetic data demonstrate how our CNN-based approach is able to remove the background noise and clearly distinguish the structures of the particle phantoms (Fig. 1), which were smeared by the noise in the low-dose measurements. These improvements are even more significant for the projections with higher noise levels.

**Figure 2:**
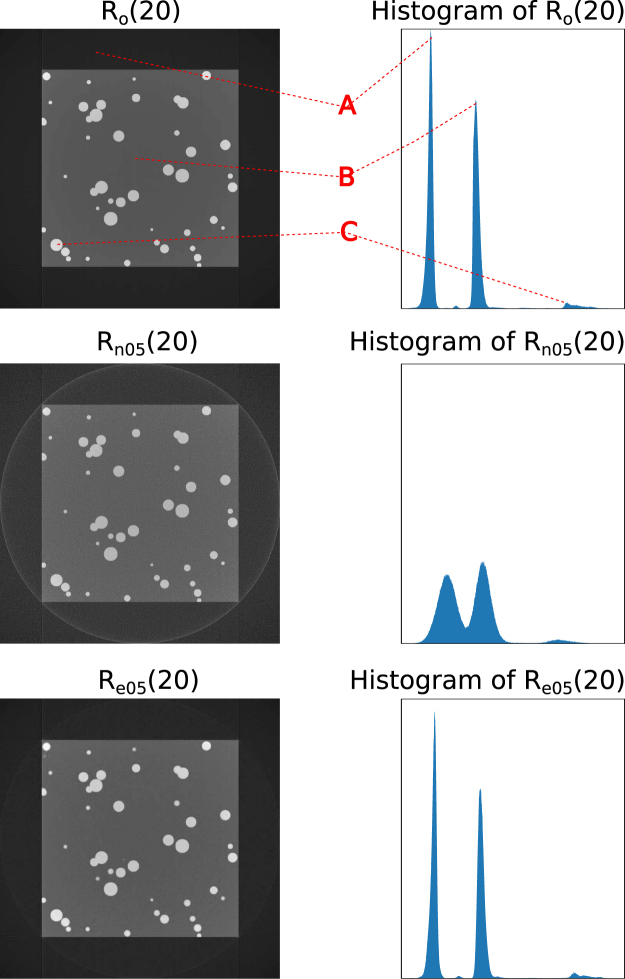
Reconstructed slice (left) and corresponding histograms (right). The top row is a slice of the ground truth reconstruction (*R*_*o*_); the middle row is the same reconstructed slice (*R*_*n*05_) from the projections with 5% noise (*P*_*n*05_); the bottom row is the same reconstructed slice (*R*_*e*05_) from the CNN enhanced projections (*P*_*e*05_). ‘A’, ‘B’ and ‘C’, correspond to the black region outside the object, the gray region inside the object and the white particles.

**Figure 3:**
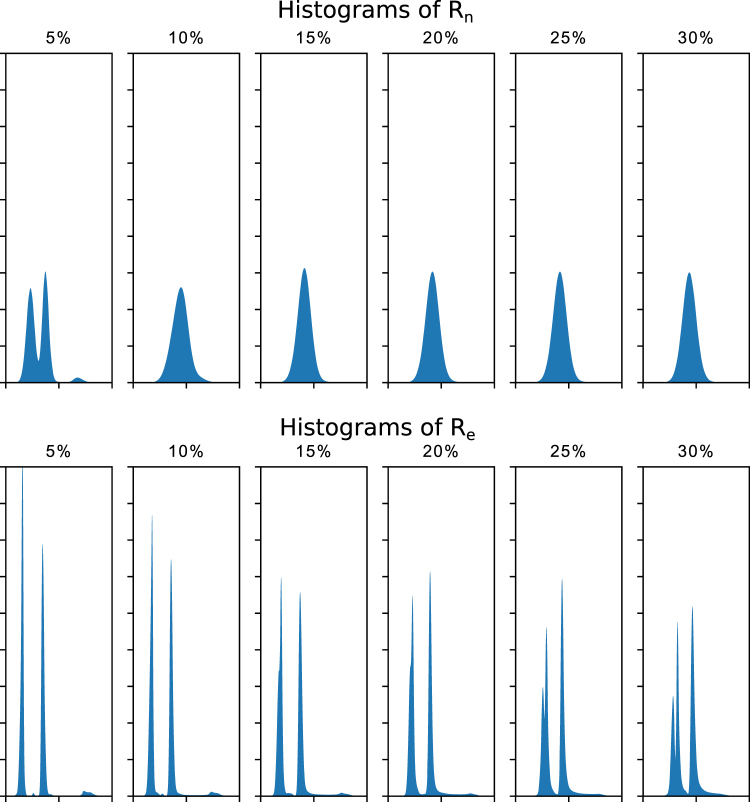
The histograms of the full 3D (512 slices) reconstructed datasets (*R*_*n*_ and *R*_*e*_) obtained from the noisy (*P*_*n*_) and from the CNN enhanced projections (*P*_*e*_).

**Figure 4:**
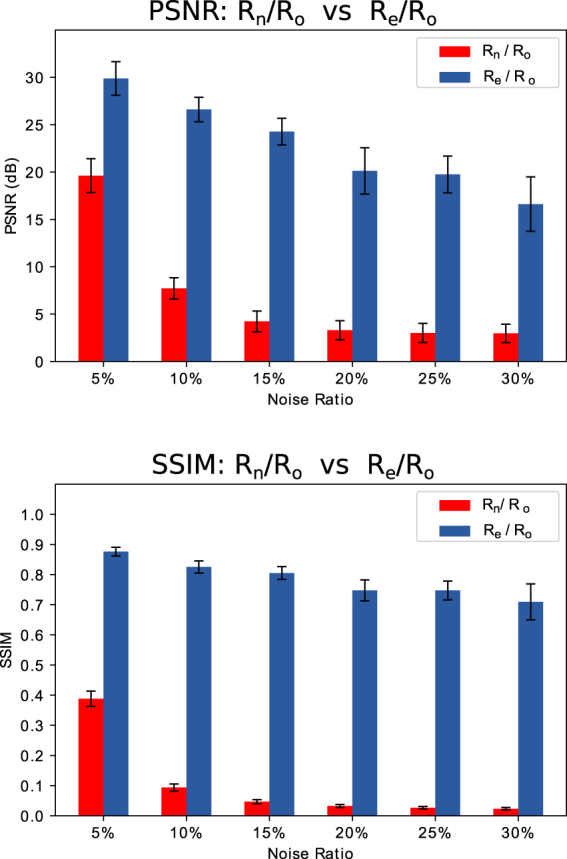
Quantitative comparison between reconstructions (*R*_*n*_ and *R*_*e*_) obtained from noisy and enhanced projections with the ground truth using Peak signal-to-noise ratio (PSNR) and Structural Similarity Index (SSIM). PSNR and SSIM are computed from the full 3D reconstructed datasets (512 slices). The higher value of PSNR and SSIM means better similarity of the compared images. The maximum value of SSIM is 1.

We performed tomographic reconstructions for *P*_*o*_, *P*_*n*_ and *P*_*e*_ to obtain the reconstructed 3D volumes *R*_*o*_, *R*_*n*_ and *R*_*e*_. We evaluated the CNN improvements by comparing image quality metrics for these 3D volumes. We first gave a direct visualization of one slice and corresponding histograms for *R*_*o*_, *R*_*n*_ and *R*_*e*_ (Fig. 2). Then we plotted the histograms of each 3D dataset for *R*_*n*_ and *R*_*e*_ (Fig. 3). At the last, we quantitatively compared *R*_*n*_ and *R*_*e*_ with the ground truth by computing two popular criteria for quantifying image quality, the Peak Signal-to-Noise Ratio (PSNR) and Structural Similarity Index (SSIM)^29^ of *R*_*n*_/*R*_*o*_ and *R*_*e*_/*R*_*o*_ (Fig. 4).

As shown in Fig. 2 (right), the image quality of *R*_*e*_ is visually close to *R*_*o*_. The corresponding histograms also proved it, see Fig. 2 (left). Compared with the ground truth (*R*_*o*_(20)), *R*_*n*_(20) shows strong noise and the three phases (A: black space; B: gray solid cubic; C: white particles) smeared in the histogram. The histogram of *R*_*e*05_(20) shows almost the same pattern as of *R*_*o*_(20). After the CNN enhancement of the noisy projections, the reconstruction quality is close to the ground truth.

The histograms of the 3D datasets *R*_*n*_ and *R*_*e*_ show significant improvements by the CNN enhancement for all the noise levels of our simulations, see Fig. 3. When the noise level is ≥10%, the histograms of *R*_*n*_ only show a single peak making segmentation by simple thresholding impossible. The histograms of *R*_*e*_ show three distinguished peaks when noise level ≤20%, and two distinguished peaks for the rest. This confirms that the CNN enhanced projections produce the reconstructions with better quality, thus making it possible to distinguish the different phases by simply thresholding.

Figure 4 shows the means and standard deviations of the PSNR and SSIM for each 3D volume. The PSNR and SSIM of *R*_*n*_/*R*_*o*_ and *R*_*e*_/*R*_*o*_ are displayed as red and blue bars, respectively. Our results demonstrate that the PSNR of *R*_*e*_/*R*_*o*_ is always much higher than *R*_*n*_/*R*_*o*_. This shows that the image quality of *R*_*e*_ is much closer to the ground truth *R*_*o*_ than *R*_*n*_. As the noise level increases, the improvements of *R*_*e*_ over *R*_*n*_ are more significant. The SSIM shows similar behavior, with even larger differences between *R*_*e*_/*R*_*o*_ and *R*_*n*_/*R*_*o*_ than observed in the PSNR. Even for the extreme noise situation (30%), the CNN can still recover enough signal to produce results with acceptable metrics (PSNR: 2.96 → 16.61, SSIM: 0.02 → 0.71). Our evaluations demonstrate that our CNN-based method produced tomographic reconstructions with consistently better quality, both in terms of their structural information and in their accuracy, even in extremely noisy situations.

### Validation on a mouse brain sample

The proposed method was further validated on a small sample of mouse somatosensory (S1) cortex containing myelinated axons. This sample has been stained with lead using a standard ROTO procedure^30^ to increase X-ray absorption contrast in the projections. After staining, the sample was embedded in plastic (EPON) to make it more X-ray resistant. After preparing the sample, it was measured with nano-CT using the TXM instrument at the 32-ID beamline of the Advanced Photon Source (APS) at Argonne National Laboratory. We first scanned the sample with 30 s exposure time (high-dose) only for 6 angles (0° to 150°, 30° per projection). We then performed a full tomographic scan (361 projections for 0° to 180°) of the sample with 2 s exposure time (low-dose) per projection. All the projections were acquired with 2*k* × 2*k* resolution and a pixel size of 30 nm. These projections were then down-sampled to 1*k* × 1*k* for subsequent processing and analysis (60 nm pixel size). The resulting reconstructions from low-dose projections mitigate beam damage, but provide limited contrast needed to resolve the width of myelin in the images.

We used the short-exposure and long-exposure projections pairs of 30° and 120° to train our CNN approach (*f*: (*P*_2_(30), *P*_2_(120)) → (*P*_30_(30), *P*_30_(120))). The trained CNN was then used to enhance all of the 361 low-dose projections. We evaluated the improvements of CNN-enhanced projections *P*_*e*_ by computing the PSNR and SSIM for *P*_*e*_/*P*_30_ and *P*_2_/*P*_30_, as shown in Table 1. The SSIM values of *P*_*e*_/*P*_30_ are much higher than the SSIM values of *P*_2_/*P*_30_. The SSIM values after CNN enhancement are about 0.9. Effectively, the CNN makes the image quality of 2 s exposure projections *P*_2_ almost equivalent to the 30 s exposure projections *P*_30_. The PSNR values also reflect higher image quality with *P*_*e*_/*P*_30_ higher than *P*_2_/*P*_30_ across the board.

**Table 1:** Comparisons of the 2 s projections and the CNN enhanced projections with 30 s projections.

Angles (degree)	SSIM		PSNR(dB)	
	*P*_2_/*P*_30_	*P*_*e*_/*P*_30_	*P*_2_/*P*_30_	*P*_*e*_/*P*_30_
0 (validating)	0.655	0.897	22.302	24.377
30 (training)	0.768	0.937	25.124	31.828
60 (validating)	0.720	0.938	27.381	34.568
90 (validating)	0.518	0.892	14.900	18.282
120 (training)	0.415	0.853	15.439	18.320
150 (validating)	0.464	0.848	12.889	25.414

**Figure 5:**
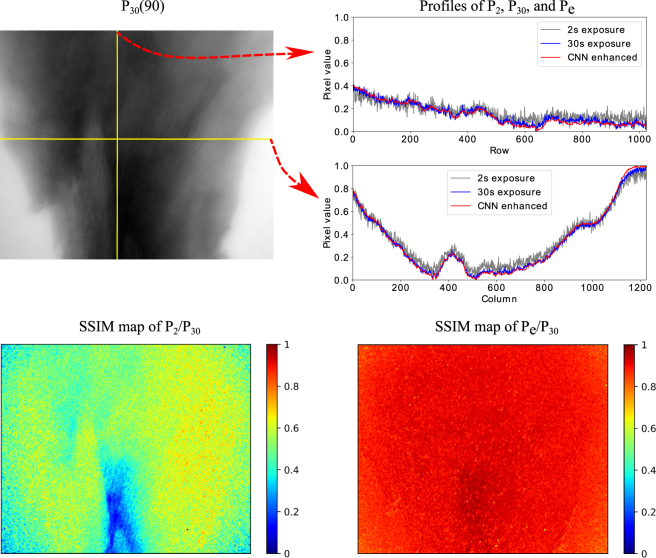
An example of the mouse brain projections with 2 s exposure time, 30 s exposure time and the enhanced projection with CNN. The top left image is the projection with 30 s exposure time at 90°. The top right are the profiles of the projections with 2 s (gray line), 30 s (blue line) exposure time, and the 2 s projection enhanced with CNN (red line). The profiles locations are as the yellow lines of the top left image. The maps of the bottom are the SSIM maps of *P*_2_(90)/*P*_30_(90) and *P*_*e*_(90)/*P*_30_(90).

We compared the details of the image quality between *P*_*e*_(90)/*P*_30_(90) and *P*_2_(90)/*P*_30_(90), shown in Fig. 5. The profiles of CNN-enhanced projection show greater overlap to the 30 s projection and much less noise than the 2 s projection. This confirms that our method provides good enhancement in both the horizontal and vertical planes of our projection images, thus providing isotropic enhancement and noise removal. When comparing the SSIM map of the 2 s projection with the 30 s projection, the majority of SSIM values range from 0.4 to 0.6; a small part near the bottom of the sample produces very small SSIM values (∼0.2). The bottom right of Fig. 5 shows the SSIM map of the CNN-enhanced projection compared with the 30 s projection. The SSIM values of the whole map are about 0.9. Most parts of the sample region have a SSIM value almost at 1. These results show dramatic improvement in the image quality by enhancing the low-dose projections with our CNN-based approach.

**Figure 6:**
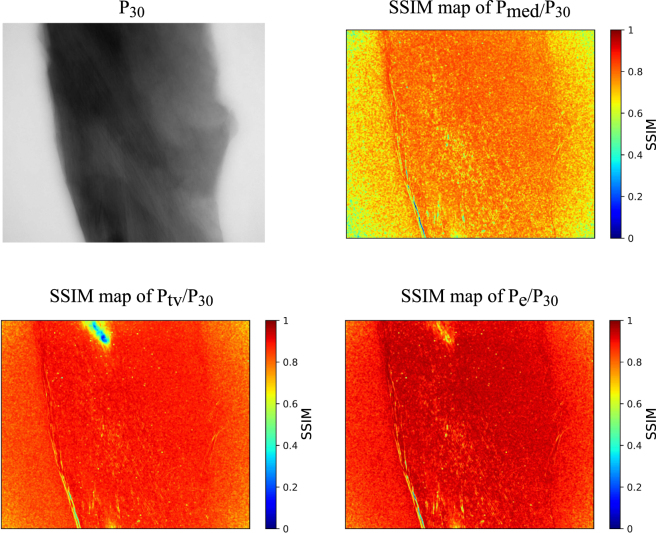
Comparing the CNN projection enhancement algorithm with the traditional denoising algorithms. The top left image is the projection with 30 s exposure time at 0°. The top right is the SSIM map between the 2 s projection denoised with median filter and the 30 s projection *P*_*med*_(0)/*P*_30_(0). The bottom left is the SSIM map between the 2 s projection denoised with tv filter and the 30 s projection *P*_*tv*_(0)/*P*_30_(0). The bottom right is the SSIM map between the CNN enhanced projection and the 30 s projection *P*_*e*_(0)/*P*_30_(0).

We also compared the CNN method with median filter and total variation regularization (TV)^31,32^, shown in Fig. 6. The SSIM values of *P*_*e*_/*P*_30_ is higher than *P*_*med*_/*P*_30_ and *P*_*tv*_/*P*_30_, where *P*_*med*_ and *P*_*tv*_ denote the projections denoised with median filter and TV regularization respectively. The CNN enhancement performs the best to recover the structural information from the noisy projection.

**Figure 7:**
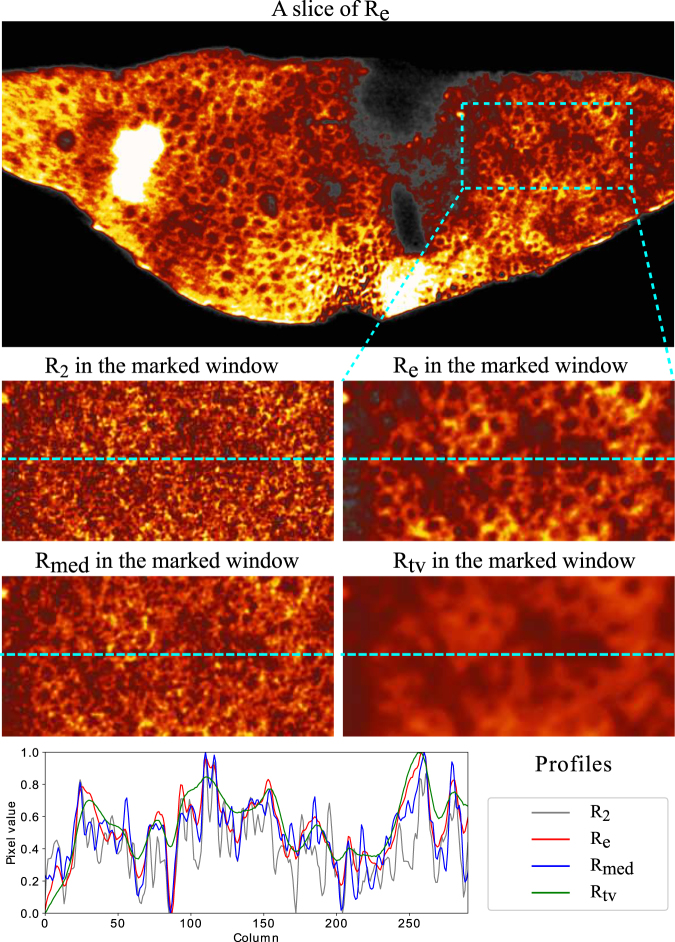
An example of the reconstructions from different quality of the projections of the mouse brain sample. The top row is a slice that reconstructed from the CNN enhanced projections. The four small images in the middle rows are different reconstructions for the section of the blue-dash rectangular window above. They are the reconstructions from the projections with 2 s exposure, the projections enhanced with CNN, the projections denoised with median filter and TV respectively. The plots in the bottom row are the profiles taken from the dash lines of the four small images.

We performed tomographic reconstructions for *P*_*e*_, *P*_2_, *P*_*med*_ and *P*_*tv*_ to obtain *R*_*e*_, *R*_2_, *R*_*med*_ and *R*_*tv*_ respectively. As shown in Fig. 7, the slice of *R*_*e*_ reveals structures that are barely visible in *R*_2_. The CNN enhancement improves the reconstruction quality for the low-dose data. On the other hand, *R*_*med*_ is rather noisy and *R*_*tv*_ is too blurry to extract the structure of the axons.

**Figure 8:**
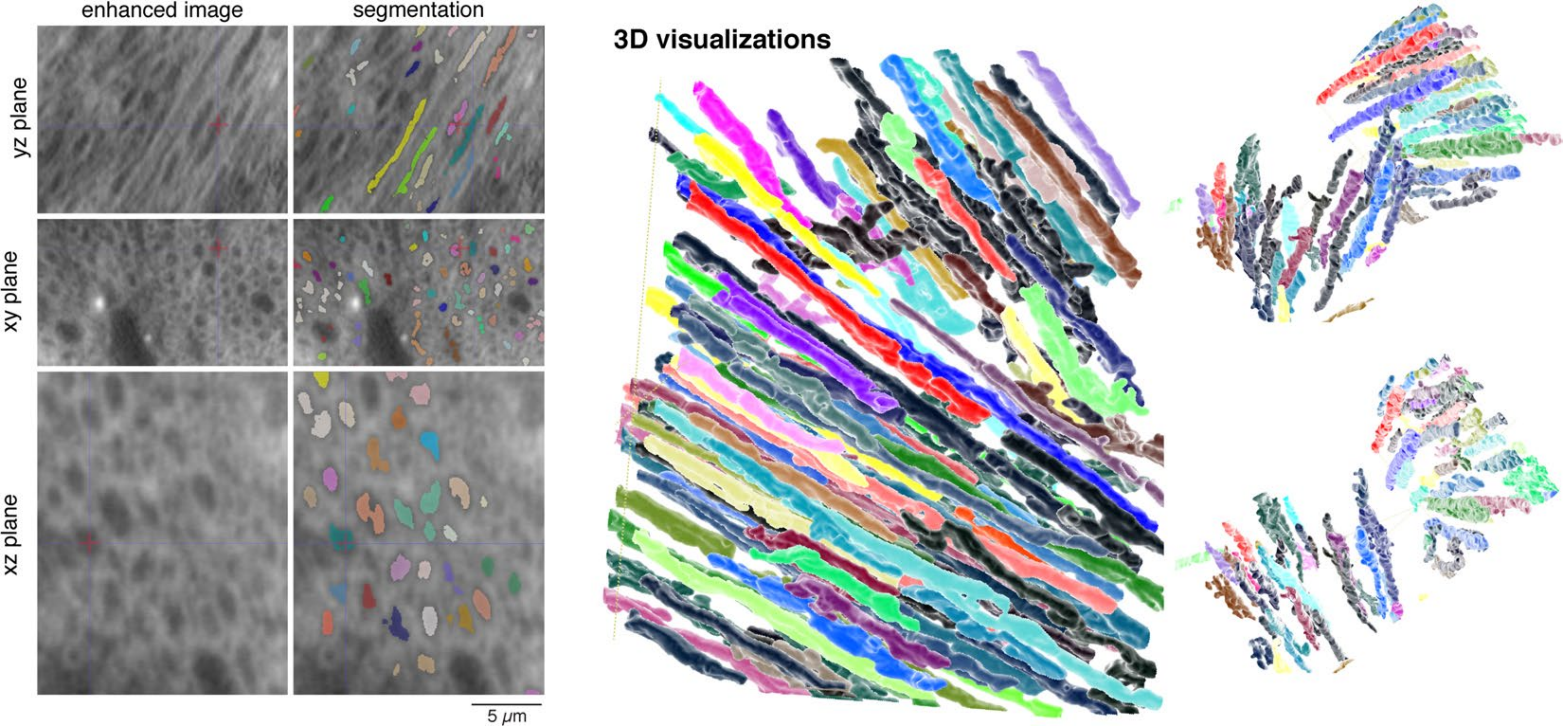
CNN-based enhancement enables automated segmentation of myelinated axons in mouse somatosensory cortex

In the CNN-enhanced volume *R*_*e*_, axons can be automatically traced over the extent of the entire sample using methods previously developed for segmenting micro-CT data^33^. The width of myelin can also be estimated in the enhanced data, as shown in Fig. 8. After enhancement, low-dose measurements can be used to estimate the thickness of myelin around the axons in the sample. In addition, tracing and resolving 2D cross-sections of axons is also improved in the CNN-enhanced images (as confirmed through manual annotations of both volumes).

## Discussion

We have introduced a new CNN-based method that dramatically reduces the exposure time by a factor of 10 for synchrotron-source X-ray tomography, while retaining structural information. Reducing imaging time decreases sample damage and provides more accurate reconstructions of the interiors of materials and biological samples. We applied this algorithm to both synthetic datasets and reconstructions of the white matter of mammalian brain samples.

As the evaluation of our CNN approach with the synthetic datasets demonstrates, the CNN does not simply ‘denoise’ the noisy projections. It also learns to distinguish the structural information of the object from the noise. The traditional denoising algorithms always lose spatial resolution if the noise is strong. However, the CNN directly learned the mapping between the noisy images and high-quality images. It can keep the resolution close to the high-quality images of learning data, when processing the tests images. The CNN processed images can be easily segmented.

Using the CNN to enhance the tomographic projections, instead of denoising the final reconstructed images^18^, was presumed to have great advantages. The tests with both the synthetic datasets and the real TXM measurements both confirmed our supposition. The CNN successfully reduces noise, and also makes the low-dose projections show structural information as well as a high-dose projection would. We also presumed fewer training images were required when the data is from the measurement of the same object as the testing data. The local features of different tomographic projections for the same object have great similarity. Despite the small size of the training datasets, they included necessary features to enhance the full-scanning projections. Thus, the accuracy of the predictions based on this training model is ensured. All our tests also proved that.

In our application of this method on a brain sample, we found that even at the low-dose that we used to obtain the results, we still observed some beam damage. Thus, at this limit, it is impossible to increase the dose further without considerable degradation. This suggests that only computational approaches can be used to improve image quality in these settings. It is only after CNN enhancement that axons could be resolved at levels sufficient to measure the thickness of myelin at high rates across the volume. Our approach for computational imaging-based enhancement promises high-resolution and dynamic imaging of brains in the future and can be combined with micro-CT approaches that capture mesoscale neuroanatomy^33^.

The running time of our CNN approach can be easily parallelized and scaled up to GPU clusters. We have tested it on the GPU cluster of Cooley from Argonne Leadership Computing Facility (ALCF). With 48 Tesla K80 dual-GPU nodes, the computing time of the data enhancement procedure can be reduced to less than 10 minutes for most of the tomographic scanning with 2 K resolution. Even including the data processing time, the whole measurement will be faster.

In addition to the good performance obtained through enhancing the low-dose tomographic projections, our CNN approach also has great potential for dynamic measurements of X-ray imaging. It speeds up the measurement process by the magnitude of 10 with keeping the data quality close to the long-time scanning. The same principle can also be applied to other X-ray imaging techniques, such as X-ray fluorescence and X-ray ptychography, to reduce dose damage or scanning time.

## Methods

### Overview of approach

Tomographic reconstructions from short-exposure X-ray images yield noisy images as too few photons are received by the sensor. The signal to noise ratio decreases as photon counts drop and the structural information about the object cannot be reconstructed successfully unless regularization methods or denoising methods are used.

Although, the features in X-ray projections of the same object for different exposure times are correlated to some extent, there is no relation readily available. This is due to the complexity of the features in the sample and variations in noise between exposure times. We used a deep CNN to learn image-to-image transformations among projections according to the rule established in the training data sets. The basic idea is based on learning a “transformation” between images taken at short (*I*_*s*_) and long (*I*_*l*_) exposures. The learning is done by minimizing the corresponding cost function,

1 $${\rm{\min }}(f({I}_{s})-{I}_{l})\mathrm{.}$$

With this basic principle, we built a CNN architecture to enhance tomographic data with short-exposure times.

### CNN architecture

**Figure 9:**
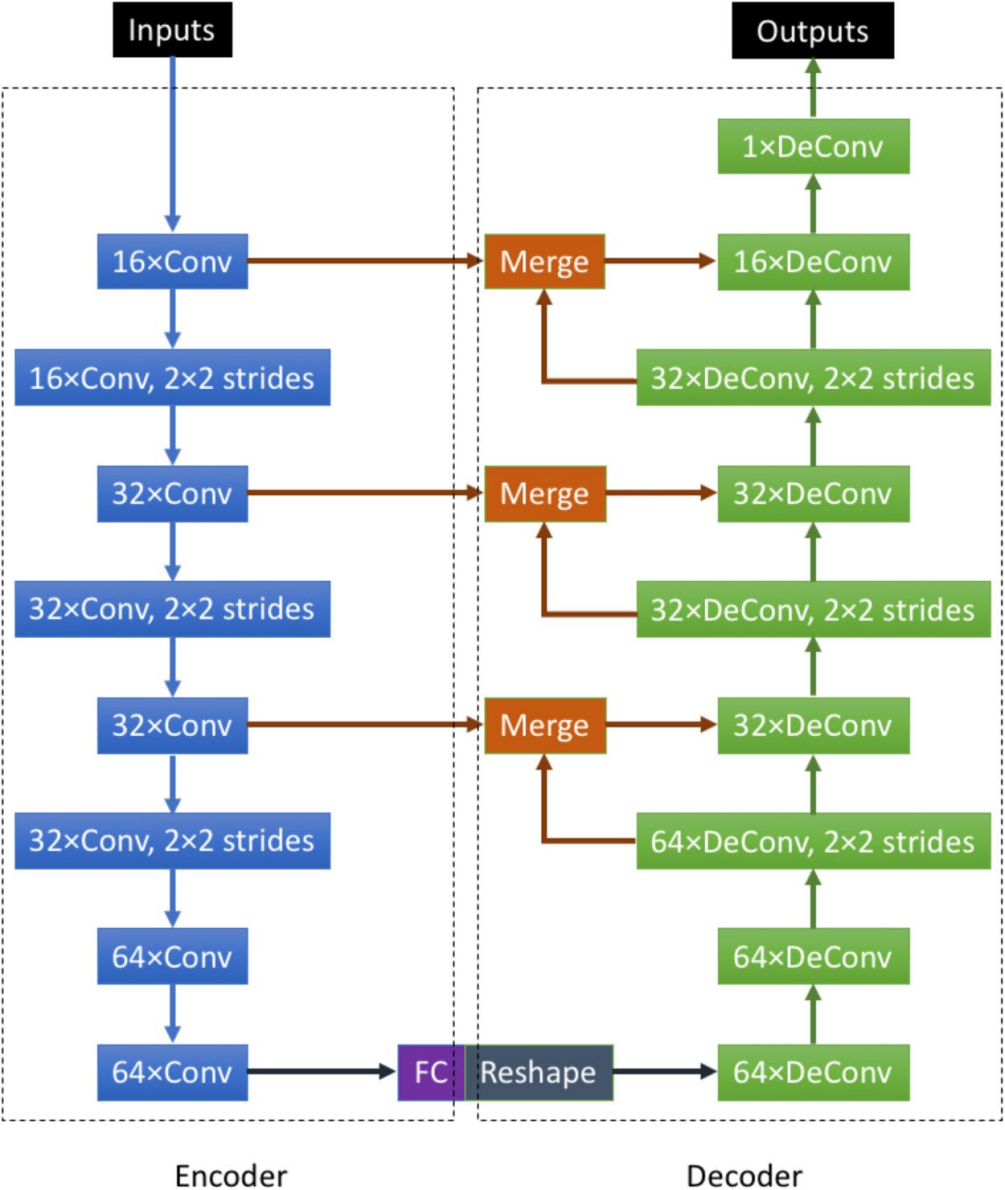
Image transformation model of CNN. “Conv” is the abbreviation of convolution kernel. “DeConv” is the abbreviation of deconvolution kernel. The numbers before the “Conv” or “DeConv” are the numbers of kernels. “FC” is the abbreviation of fully connected layer. All the convolutional and deconvolutional layers use 3 × 3 kernels. All the layers use Relu as the activation functions^43^.

The proposed CNN architecture was inspired by various image transformation models in literature^27,34^. The input and output of the network are both images, instead of images as inputs and scalar labels as outputs for the typical CNN model^35^. As shown in Fig. 9, the network architecture consists of two principal parts: the image encoder and the data decoder. The image encoder is composed of 8 convolutional layers and a fully connected layer. All convolutional layers use 3 × 3 convolution kernels. We increase the number of convolution kernels for each layer from 16 to 64. Three of the convolutional layers use a 2 × 2 strides to compute the convolution from every 2 pixels of the corresponding images^36^. This reduces the image width and height by half after the convolution. The convolutional layers with and without strides extract multiple features of the input images (*W* × *H*) from different scales (*W* × *H* to *W*/8 × *H*/8). Together with the fully connected layer, the network enforces image information to be sparse with various feature maps. The encoder aims to fit the image information with specific target data.

The second half of the network is the decoder. The encoded data are reshaped to a new image size of (*W*/8 × *H*/8). We use 9 deconvolutional layers^37^, which are also called transpose convolutional layers. All deconvolutional layers use 3 × 3 deconvolution kernels. We decrease the number of deconvolution kernels for each layer from 64 to 1. Three of the deconvolutional layers use the 2 × 2 strides, which double the image width and height. These decovolutional with and without strides generate layers of feature maps with different scales. At the end, we use a deconvolutional layer only with one deconvolutional kernel to generate a single output image that matches the target image. Using the deconvolutional layers as the decoder has a fundamental advantage of keeping the image with good resolution. This avoids smoothing the patterns and boundaries of structures by using convolutional layers. The main reason is that the convolutional layers compute the pixels of the new image from each of their 3 × 3 neighbors. Deconvolution is the inverse of this process and can make the output images as sharp as the input images.

Using merge layers is another important way of making the output images sharp. In the decoder part of the network, feature maps generated from the deconvolutional layers are concatenated with the preceding feature maps of the same scale from the encoder part. The proportions of the merged feature maps from different layers are decided by the kernel number of these layers, see Fig. 9. With these merged layers, the feature maps of the decoder part include the features with different resolutions of the input images, including the original resolution of the input images. They can avoid the resolution loss during the down-sampling process of the encoder.

The objective function of the CNN is computed based on the peak signal-to-noise ratio (PSNR):

2 $${\rm{\min }}(\frac{1}{PSNR})\mathrm{.}$$

The PSNR can be calculated as:

3 $$PSNR=10\cdot {\mathrm{log}}_{10}(\frac{{I}_{max}}{MSE(f({I}_{s})-{I}_{l})}),$$

where *I*_*max*_ is the possible maximum pixel value, *MSE* is the mean square error, and *f*(*I*_*s*_) is the new image generated by the CNN.

### Normalization and patch extraction

To infer high fidelity reconstructions from short-exposure tomographic measurements, we trained a deep CNN to learn the mapping between the two conditions. To prepare the training data, we first scanned the object for a few angles for obtaining images taken at relatively long-exposure times (*I*_*l*_). Then, we performed full-angle tomographic scanning to obtain projections with shorter exposure times (*I*_*s*_). We used the long-exposure time projection and its related short-exposure time projection as the training data to fit the CNN:

4 $$f\,:{I}_{s}(a)\,\to \,{I}_{l}(a),$$

where *I*_*s*_(*a*) and *I*_*l*_(*a*) are the projections of angle *a*. Normally, two or three angles of projections are enough for training.

We processed the training data in two steps before tomographic reconstruction: normalization and patches extraction. The normalization was done in two steps: ${I}_{1}=\frac{I-\overline{I}}{\sigma (I)}$ and ${I}_{2}=\frac{{I}_{1}-\,\min ({I}_{1})}{\max ({I}_{1})-\,\min ({I}_{1})}$. We extracted overlapped small patches from these normalized images as the final training inputs and outputs using the same approach described in^35^.

The reason and benefit to use the small patches are as following:

- Every projection of the same object shows different patterns. We only had one angle view of the image and this was not sufficient to predict all the projections. However, if we extracted small patches from the projections, we could usually find very similar local features from these patches. Thus, the patches from one projection include enough local features to train the network and to predict the rest of the projections.
- The overlapped patches increase the number of the training data from a few projection images to ∼10^5^ of small images. The distances between the data points become smaller than directly using the full images. This can significantly reduce the probability of overfitting.

After we trained the CNN transformation model, we can save the fitted weights of the network to predict projections with quality close to that of the long-time projections (*I*_*l*_) from the short-exposure-time projections (*I*_*s*_). Small patches with the same size as the training data were extracted from these projections (*I*_*s*_) and used as the input for the trained CNN. We reconstructed these patches to obtain enhanced projections. These enhanced projections were used for the tomographic reconstruction to get the final result.

### Tomographic acquisition and reconstruction

The synthetic projections were generated from the simulated phantoms with Radon transform^38^, which is an integral transform whose inverse is used to reconstruct images from X-ray CT scans. The experimental dataset was collected at the Transmission X-ray Microscope (TXM) of sector 32-ID at the Advanced Photon Source^2^ and formatted in Scientific Data Exchange standard^39^. We performed tomographic reconstruction with the open-source TomoPy toolbox^31^ using the CNN enhanced short-exposure projections and the long-exposure projections. We applied a Fourier grid algorithm (GridRec) with a Parzen filter for reconstruction^40^, because of its good balance of the reconstruction speed and accuracy, however other tomographic reconstruction methods can as well be used.

### Axonal segmentation

After reconstructing images of a small mouse brain sample, we applied segmentation methods previously used to segment blood vessels and cell bodies in micro-CT data^33^ to segment myelinated axons in the sample. To do this, we first trained a random forest classifier using an interactive tool for segmentation called ilastik^41^ using a combination of edge, texture, and intensity-based 3D features. Following training, we applied the classifier to a small reconstructed image volume from a cube of mouse cortex (234 × 400 × 300). We then thresholded the classifier probability estimates (threshold = 0.92) and applied 3D morphological filtering operations to the resulting thresholded data (see^33^ for more details). Finally, we visualized the segmented data using the multi-view projection and 3D visualization tool in ITK-Snap^42^.
